# Community Mental Health Workers' Experiences of Care‐Coordinating Hospital Discharge of Patients With Diagnosis of Mental Health Condition Into Community Mental Health Integrated Services in the Northwest of England

**DOI:** 10.1111/inm.70334

**Published:** 2026-10-01

**Authors:** Samuel Ndoro, Guillermo Perez Algorta, Caroline Swarbrick

**Affiliations:** ^1^ Division of Health Research Lancaster University Lancaster UK

**Keywords:** care coordination, community mental health services, community mental health workers, experiences, hospital discharge, mental health conditions, patients, qualitative inquiry

## Abstract

The significance of community mental health workers in coordinating hospital discharge of patients with diagnosed mental health conditions is globally recognised, yet research on their experiences is scarce. This study contributes to knowledge through a qualitative empirical intrinsic case study of community mental health workers' experiences. This study aimed to explore community mental health workers' experiences of care‐coordinating patients diagnosed with mental health conditions discharged from the hospital into community mental health integrated services. Guided by constructivist inquiry, the qualitative study focused on co‐constructing meaning to understand community mental health workers' experiences in care coordination. Two theoretical lenses informed the study: Ecological Systems Theory, to understand multi‐level influences, and Normalisation Process Theory, to explore how care coordination practices are embedded in discharge processes. Fourteen community mental health workers aged 18 and above were recruited from one NHS Trust in Northwest England from October 2021 to June 2022. Data were collected through semi‐structured interviews, audio‐recorded, transcribed verbatim, and analysed using reflective thematic analysis, generating themes and subthemes. Analysis identified 13 subthemes grouped into four major themes: (1) Experiences of collaborative working; (2) Challenges and enablers to care coordination; (3) Experiences of organisational system and culture; and (4) Community Mental Health Workers' personal experiences of professional growth. Findings highlight poor communication, staff shortages, high caseloads, limited community mental health services, and challenges in care coordination. The study provides valuable insights for improving community mental health workers' clinical practice and informs future research and policy development in community mental health care.

## 1 Introduction

Globally, care coordination for patients with mental health conditions transitioning into Community Mental Health Integrated Services (CMHISs) remains challenging (Gandre et al. 2020). Barriers include poor communication among multidisciplinary teams (MDTs) and insufficient Community Mental Health Workers (CMHWs) to follow up post‐discharge, increasing the risk of patient relapse and readmission (Thornicroft and Tansella 2013; World Health Organisation 2018, 2021). Although there is broader literature on care coordination, discharge planning, and the systemic challenges facing the community mental health workers, this study aims to extend it by offering a more granular, context‐specific account of how these pressures are negotiated in everyday community mental health practice. Addressing these gaps is critical to improving continuity of care and patient outcomes in community mental health services.

## 2 Background

In the UK, implementing care coordination remains challenging due to difficulties in collaborative working and ongoing workforce limitations. A key issue is the shortage of community mental health workers (CMHWs) and fragmentation within community mental health integrated services (CMHISs), which increases caseloads, reduces service availability, and contributes to staff burnout (Gandre et al. 2020; Liberati et al. 2021). Fragmented services and weak integration with wider health and social care systems further disrupt continuity of care (Tracy et al. 2019). Addressing these challenges is essential to optimise the effectiveness of CMHWs and ensure equitable access to community mental healthcare (National Institute of Clinical Excellence 2016).

Furthermore, in the UK, CMHWs include community mental health nurses and community psychiatric nurses (CPNs), occupational therapists (OTs), social workers (SoWs), psychologists, support workers (SWs), and psychiatrists (Schadewaldt et al. 2014). Their responsibilities range from early discharge planning to regular MDT reviews involving patients and carers, as well as identifying social needs such as housing and support networks. CMHWs play a key role in preparing patients and families for discharge through education and collaborative planning, whether discharge occurs under the Mental Health Act 1983 (as amended in 2025) or on a voluntary basis, with continued post‐discharge monitoring and treatment to support stability and reduce relapse risk.

Care coordination supports transitions and continuity of care for people with mental health conditions through collaborative working across CMHISs (van Veen et al. 2020). The transition from hospital to community care is a particularly vulnerable period that requires comprehensive planning to reduce the risk of relapse (Care Quality Commission 2024). During this phase, CMHWs manage multiple responsibilities and collaborate with a wide range of providers (Simpson et al. 2009). Their work includes case management, multidisciplinary team (MDT) collaboration to ensure continuity of care, arranging home and clinic appointments, and conducting follow‐up visits (Williams and Smith 2019).

CMHWs support individuals with a broad range of mental health needs, including psychosis, schizophrenia, anxiety, depression, personality disorders, suicidal ideation, self‐harm, eating disorders, and substance or alcohol misuse (American Psychiatric Association 2013; National Institute of Clinical Excellence 2011). They also address comorbid physical health conditions such as diabetes, hypertension, and heart disease (Butcher et al. 2020; Clarke and Walsh 2009; Department of Health 2011). Their responsibilities include comprehensive risk assessment, interdisciplinary care planning, and the delivery of evidence‐based interventions such as counselling, psychoeducation, treatment, and crisis support (Department of Health 2014; Radley et al. 2021).

Within MDTs, CMHWs fulfil multiple roles in supporting patients with mental health diagnoses; however, poor communication between community mental health teams (CMHTs) can delay timely care during transitions to the community (Parliamentary and Health Service Public Ombudsman 2024). This study was conducted at a critical time, amid increasing pressure to strengthen community care coordination in response to National Health Services (NHS) challenges, including hospital capacity shortages, workforce retention issues, and systemic failures in service delivery (British Medical Association 2024; Ham 2023; House of Commons Health and Social Care 2019).

## 3 Research Question

This study aimed to answer the research question: What are community mental health workers' experiences of care‐coordinating discharge of patients with diagnosed mental health conditions into community mental health integrated services?

## 4 Methods

### 4.1 Epistemological and Ontological Position

This study adopts a constructivist epistemological and ontological stance, which assumes that meaning is actively constructed through individuals' experiences within specific social and relational contexts, giving rise to multiple realities (Byrne 2022). Epistemologically, the study acknowledges knowledge as subjective, contextually embedded, and co‐constructed through interaction between the researcher and participants during the process of coordinating hospital discharge (Bagnoli 2013). Consistent with this position, a qualitative methodology was employed to enable an in‐depth exploration of participants lived experiences and the interpretive processes through which meaning is formed. The flexibility of semi‐structured interviews supported the application of a constructivist approach by allowing meanings to be generated and interpreted collaboratively. Overall, the study's ontological and epistemological assumptions align with a qualitative methodology, which provides the conceptual framework guiding knowledge production, while the selected methods represent the practical techniques used to generate that knowledge (Durham et al. 2015).

### 4.2 Theoretical Underpinnings of This Study

This study's findings are underpinned by Ecological Systems Theory EST and Normalisation Process Theory (NPT). EST offers a way of understanding how practitioners' experiences of care coordination are shaped by multiple interacting system levels, from immediate team dynamics (microsystem) to organisational structures and national policy environments (macrosystem). In this study, EST helps illuminate how pressures at different system layers such as staffing constraints, interagency relationships, and policy expectations combine to influence everyday discharge practices. Normalisation Process Theory (NPT) complements this by explaining the mechanisms through which new or complex practices become embedded in routine work, particularly through constructs such as coherence (how staff make sense of their role) and collective action (the operational work required to enact it). Applying NPT enables us to interpret participants' accounts not just as descriptions of challenges, but as insights into why certain aspects of care coordination fail to become fully normalised within services. Together, EST and NPT provide a coherent theoretical lens that links individual experiences to wider structural forces and the processes that support or hinder the embedding of collaborative discharge practices.

### 4.3 Study Design

A qualitative methodology study which used semi‐structured interviews. Semi‐structured interviews were selected because they offer the flexibility needed to explore practitioners' experiences in depth while still providing enough structure to ensure coverage of key areas related to care coordination and discharge processes. It enabled participants to elaborate on complex relational, organisational, and emotional aspects of their work insights that would have been difficult to capture through more structured or survey‐based approaches.

### 4.4 Ethical Considerations

This research received ethical approval from the Lancaster University Ethics Committee and the NHS Integrated Research Application System (IRAS) 291320. Data were disposed according to the data protection policy of the university. Insider status provided leverage for deep contextual understanding and an established rapport with participants.

### 4.5 Recruitment and Sampling of Participants

Purposive sampling was used from October 2021 to June 2022. Participants were CMHWs aged 18–65 from one geographical area in England who had worked in CMHTs for over a year, spoke English, and provided informed consent. Participant information sheets were emailed to potential participants via secure Trust accounts, with contact made through existing professional links. Recruitment materials were advertised on the NHS Trust intranet. Interested participants contacted the chief investigator and eligible participants were provided with study participant information. Fourteen participants gave consent to take part. Participation was voluntary, with individuals given at least 24 h to review study information and ask questions before consenting (Offredy and Vickers 2013). Additionally, it was acknowledged that sample size in research varies depending on the type of study. It is still up for debate how large a sample size is appropriate for qualitative research (Hennink and Kaiser 2022; Young and Casey 2018). In qualitative research, sample sizes tend to be smaller compared to quantitative studies (Vasileiou et al. 2018) as qualitative research focuses on in‐depth exploration and understanding of phenomena, rather than statistical generalisation (Sandelowski 2000). Sample size was not viewed as a limitation in itself; instead, the value was placed on the depth and richness of the data collected and the insights gained from thorough analysis (Emmel 2013).

### 4.6 Data Collection Procedure

Data were collected through semi‐structured interviews with consenting participants, conducted via Microsoft Teams or face‐to‐face using probing questions when needed (Saarijärvi and Bratt 2021). The researcher kept a reflective diary. Interviews, averaging 25 min, were recorded on Microsoft Teams and a digital hand‐held device. All recordings, transcripts, and consent forms were anonymised and stored securely on the University's OneDrive. The lead author transcribed the data verbatim and imported the transcripts into Atlas.ti (version 24) for coding and data management.

### 4.7 Findings

Participants provided written consent with *n* = 14. Most participants were British women, and participants were drawn from a range of job roles. The demographic details presented in Table 1 (using pseudonyms) provide an overview of the participants' characteristics, enabling a better understanding of the participant group composition.

**TABLE 1:** Participants' table of demographic characteristics.

Characteristic	Job role	Participants
		*N* = 14
Professional role	Senior Mental Health Practitioner	3
	Team Manager	2
	Occupational Therapist	2
	Community Psychiatric Nurse/Care Coordinator	2
	Social Worker/Care Coordinator	1
	Consultant Psychiatrist	3
	Pharmacist	1
Gender	Female	10
	Male	4
Ethnic background	White British	9
	Black British (African)	3
	British Asian	1
	Other/Not Stated	1
Years working in CMHS	0–1 year	0
	2–5 years	2
	6–10 years	2
	11+ years	10

Table 1 shows that six of the 14 participants who provided information about their work experience had an average of 10.8 years of work experience. Additionally, five of the participants had experience in dual roles as care coordinators.

In terms of the interview process, 10 were conducted via Microsoft Teams, and four were conducted face‐to‐face. These demographic details provide an overview of the participants' characteristics, enabling a better understanding of the study's sample composition.

### 4.8 Data Analysis

This qualitative inquiry study used Reflective Thematic Analysis (RTA) to explore CMHWs' experiences in coordinating hospital discharge for mental health patients. RTA aligns with the study's constructivist assumptions, supporting subjective, interpretive analysis consistent with its ontological and epistemological paradigms (Byrne 2022). Reflective thematic analysis (RTA) was appropriate because of its inductive process (Braun et al. 2023). RTA enabled the researcher to gain an in‐depth understanding of the collective or shared meanings and experiences of CMHWs' experiences of the care‐coordinating hospital discharge process The study utilised (Braun and Clarke 2006, 2022, 2023, 2024)'s six steps of reflective thematic analysis. First data familiarisation to theme construction, with explicit development from codes to themes. Interview transcripts were repeatedly read to achieve deep immersion with data, during which salient ideas, meanings, and patterns were noted. Initial codes were developed through open coding, both manually and using Atlas.ti, whereby meaningful data segments were labelled to capture features such as recurrent experiences, actions, or perspectives. These initial codes were then compared and grouped through constant comparison, enabling conceptually related codes to be grouped into candidate themes. Atlas.ti's retrieval, search, and network functions supported the analysis of relationships between codes, the refinement of thematic boundaries, and the identification of subthemes. Themes were iteratively reviewed to ensure internal coherence and distinctiveness, then clearly defined and named to reflect their central organising concepts. Throughout the process, analytic memos and annotations were used to document reflective decisions and justify theme development, enhancing transparency and rigour. The final stage involved integrating the fully defined themes into a coherent analytic account, supported by illustrative diagram with themes and subthemes. Interview excepts were presented, supported by demonstrating the progression from raw data to codes, themes, and reported findings. Reflexive TA does not seek code saturation (Braun and Clarke 2021). In addition, in line with RTA, this study did not aim for code saturation. Instead, assessing ‘meaning saturation’, the point at which additional interviews no longer generated new conceptual insights (Hennink and Kaiser 2022).

## 5 Researcher Positionality and Reflexivity

The researcher's background as a senior mental health nurse and PhD post graduate researcher with experience in care coordinating hospital discharge of patients with a diagnosis of mental illness shaped both the design of this study and the interpretation of the data. Awareness of this positionality was crucial throughout the research process, prompting careful reflection on how prior knowledge, values, and assumptions could influence interactions with participants and analytic decisions. The researcher maintained a reflective journal throughout data collection and analysis to document thoughts, emotional responses, and emerging interpretations, providing a mechanism to critically examine potential biases and preconceptions. Additionally, the researcher engaged in ongoing discussions with academic supervisors to challenge interpretive assumptions and ensure that participants' voices remained central to the analysis and consensus was reached.

## 6 Reflective Thematic Analysis

After completing the reflective thematic analysis and familiarisation with the data according to Braun and Clarke (2024), 14 interview transcripts were transported into Atlas.ti 24 software. From these interview transcripts 1034 were developed, and 124 codes were organised into 14 categories. During the coding phase, relevant data segments connected to the research questions were manually labelled, incorporating reflexive engagement to recognise the impact of the researcher's viewpoint. The codes were subsequently grouped into possible categories to develop themes on conceptual similarity and frequency in participants' quotations. By employing an iterative approach to revise and improve, key themes and subthemes were developed to encapsulate both common trends and differences found in the data. The ultimate reflective thematic analysis was shaped by both semantic and latent content, intending to reflect the intricacy of the participants' experiences while staying anchored in the data (Braun and Clarke 2024). Four main Themes and subthemes were developed from data. (1) Experiences of collaborative working, (2) Challenges and enablers to care coordination, (3) Experiences of organisational system and culture, (4) CMHWs' personal experiences of professional growth (illustrated in Figure 1).

**FIGURE 1:**
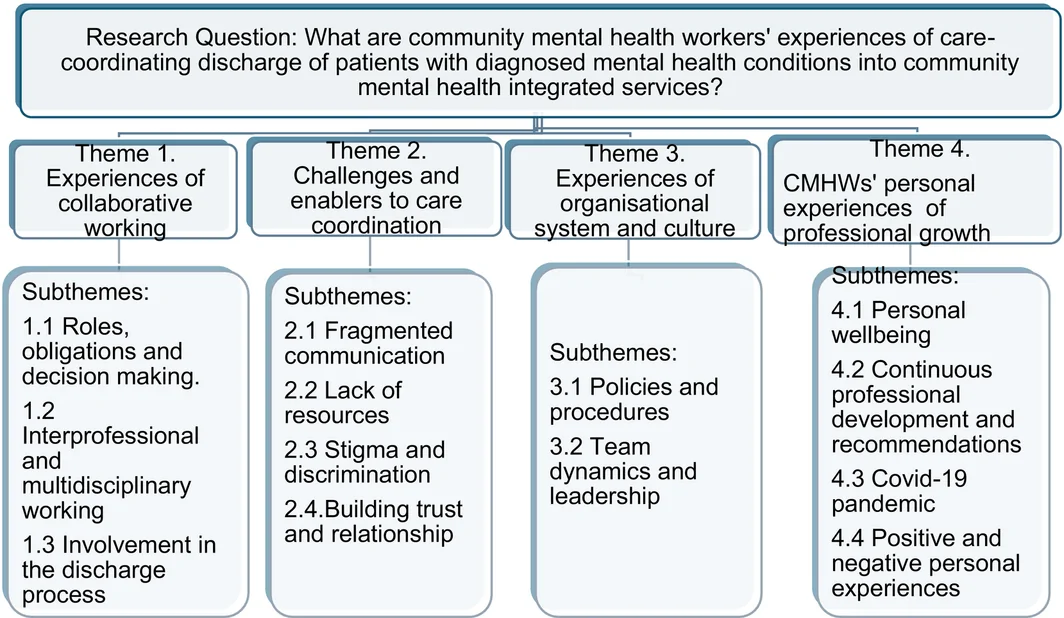
Themes and subthemes identified across the fourteen interviews.

Excerpts from participants' interviews are used to illustrate each theme and subtheme. Textual omissions are shown using ‘[…]’. The term ‘sic’ shows that the quoted text is reproduced exactly as it appeared in the original source, including any mistakes or errors, and the clarification is provided to acknowledge and address the mistake within the quote.

### 6.1 Theme 1. Experiences of Collaborative Working

Collaborative working significantly influences the experiences of CMHWs, particularly through interactions and coordination among various CMHSs. Effective collaboration refers to facilitated communication among MDTs and shared decision‐making processes, which collectively enhance the provision of care of patients in the community. There are three subthemes to this theme: roles obligations and decision‐making, and interprofessional and multidisciplinary team working and involvement in the discharge process.

#### 6.1.1 Subtheme 1.1. Roles, Obligations, and Decision Making

The first subtheme roles, obligations, and decision‐making showed that CMHWs had diverse experiences in their roles and responsibilities, with a notable emphasis on the significant aspect of decision‐making during care‐coordination of patient hospital discharge.

Many CMHWs expressed their experiences of duties and responsibilities when collaborating with community mental health teams and other organisations. They emphasised the need for effective communication, shared decision‐making, and coordination of patient hospital discharge among CMHWs, including doctors, nurses, pharmacists, social workers, and other members of the multidisciplinary team (MDT). One CMHW shared his experience of care‐coordinating patient hospital discharge, outlining his primary responsibilities, which include risk assessment, risk management, and planning for patients:

My duties and responsibilities involve mainly risk assessment as well as managing risk as well as planning […]. In terms of treatment discussing with the consultant and other teams and if there are some things like medication review and other psychological treatment required. [Bongani]

Whereas another CMHW recognised the importance of decision‐making in MDT, valuing input from and experiences of working with CMHWs during patients' hospital discharge, saying:

Well, I suppose I am the consultant, so ultimately, it's my decision whether the patient is ready to go home or not but obviously, we'll make decisions as an MDT and we discuss how the nurses are feeling about the patient and what I'm thinking, what other people are thinking, what the patient is thinking […]. [Jessica]

In addition, the majority of CMHWs highlighted the holistic and varied nature of their role, involving risk assessment, therapeutic interventions, provision of equipment, social inclusion, work‐graded exposure, crisis management, safety planning, and psychoeducation:

So, my duties and responsibilities involve assessing risk and ensuring that the patient has … It is risk is monitored, but in addition to that, you also provide therapeutic interventions in the community as well … And it's quite holistically and it's quite varied at the OT role within the community Home Treatment Team. [Julie]

Similarly, one CMHW shared her experiences of participating in the discharge process and contributing to decision‐making for future arrangements prior to a patient's hospital discharge, saying:

My involvement in the discharge process is attending ward rounds to develop a plan with the patient and the ward team, attending zoning at the Early Intervention Team to update the Multidisciplinary Team and put the plan, establish different plans and then act on them and then update the team and then it might be helping the patient with practical things when they're an inpatient. [Kate]

This quotation captures a variety of cases met by the CMHWs when making referrals of patients to other services. Furthermore, another CMHW highlighted the complexity of her duties as a care coordinator saying:

Yes, my duties are to function as care coordinator to a designated number of people. My caseload now stands at 46 people. I will review their care packages, I will monitor their mental health, I'll liaise with different agencies, […] do some Care Programme Approach (CPA) work and as a clinician I also do medication administration and help other teams with their clinical duties. [Rose]

This quote highlights the multifaceted role of the community mental health workers, engaging in both clinical duties, such as medication administration and helping other teams, while also being involved in Care Programme Approach (CPA) work, illustrating their multiple roles and dedication within CMHTs. CMHWs face a range of complex responsibilities as part of their role; for example, establishing therapeutic relationships with patients and offering assistance throughout the process of coordinating patient discharge from the hospital.

#### 6.1.2 Subtheme 1.2. Interprofessional and Multidisciplinary Team Working

In addition, the majority of CMHWs highlighted the holistic and varied nature of their roles and responsibilities, involving risk assessment, therapeutic interventions, provision of equipment, social inclusion, work‐graded exposure, crisis management, safety planning, and psychoeducation. For example, CMHWs highlighted their experiences of interprofessional and collaborative working with MDT:

So, my responsibility, I am a senior mental health practitioner with the XY Team […]. And my responsibility is to work as part of a team, which is essentially facilitating potential discharge, or early discharge, or supported discharge […] and in the process work with the MDT. So that would consist of us as a team, the consultant, the ward staff, to liaise in that process. [Richard]

Some CMHWs described their experience of involvement in the discharge process with MDT by arranging for patients to have a Section 17 home leave (under the Mental Health Act) for an agreed period:

Yeah, and then when at a point whereby the patient is going to be gradually discharged, which means their consultant want them to go on Section 17, leave for a week, and let us monitor how things are going at home. I will do the home visit […] and then continue to monitor them until other services takes over. [Bongani]

Similarly, other CMHWs shared their experience in working within MDTs, noting that, when a patient is admitted to an acute ward, the care planning process should already consider the discharge plan as part of the overall pathway, starting from the moment of admission:

My understanding is that when a patient is admitted onto an acute ward, part of their care planning pathway is about what I'm going to do on discharge, they should be planning their discharge from the point of admission. [John]

In addition, one CMHW narrated the experience in her role with the Care Programme Approach (CPA) and MDT, particularly focusing on discharge planning when patients are leaving specialist hospitals, for example:

Oh, my dealings with the Care Programmed Approach (CPA) tends to be on discharge from when patients are discharged from specialist hospitals. So, I will get involved in CPA meetings which are usually every three months for patients who are in hospital settings […]. We deal with patients who are established in the community, and they've got support and care packages. [Rose]

#### 6.1.3 Subtheme 1.3. Involvement in the Discharge Process

Another important aspect explored relates to how CMHWs are engaged in patients leaving the hospital, known as the discharge process. This subtheme examines various aspects of how CMHWs participate and contribute during the discharge process. This subtheme examines various aspects of how CMHWs participate and contribute during the discharge process, highlighting the importance of multidisciplinary teamwork and shared responsibilities during care‐coordinating patient hospital discharge, for example:

Yes, certainly, […], so I often get involved when I was on the ward, I used to get involved in a lot of discharge planning and I would attend the ward rounds and sometimes I'd be asked to do a home visit assessment and see if that person needs a package of care before they return […]. [Julie]

The reported value of involvement in care‐coordinating patient hospital discharge was described by CMHWs who shared their experiences. One CMHW described his experience of participating in patient discharge meetings to assess patients for supported discharge finding those suitable for the service:

We could attend two or three business meetings a week to screen all the patients for supported discharge […]. And kind of show them that once they're discharged from the ward there is something on offer and they will be supported. Which in time and the length of stay in hospital is shorter. [John]

Similarly, some CMHWs narrated their experiences, explaining that patients are gradually prepared for discharge by first assessing their improvement and managing risks in the community. This involves testing with supervised leaves and progressively transitioning to unsupervised and longer periods, such as day or overnight leave:

…when we all feel that they have improved to a significant level and the risks could be manageable in the community then we start testing out with leaves […]. And if everything goes well, yes, they can be discharged into the community […]. Otherwise, we discharge them with the support of the Home Treatment Team. [Naresh]

The CMHWs' experience highlights the importance of ensuring proper medication management and discharge planning to avoid potential complications for patients:

Recently we actually had a patient who was discharged. They were on Clozapine, but I don't know what happened in that process, but they seem to have been sent home without enough supply of their Clozapine. So of course, Clozapine, if you miss it for 48 h then you need to be retitrated. And at the moment most of our retitrations are taking place in hospital, so that would require for the patient to be readmitted unnecessarily, if I may say that […]. [Rudo]

Consequently, theme one explored experiences of CMHWs' roles, obligations and decision‐making, and interprofessional and collaborative working involved in the discharge process. CMHWs shared their valued experiences about their interprofessional and collaborative working involvement in the discharge process to ensure patient‐centred care.

### 6.2 Theme 2. Challenges and Enablers to Care Coordination

Theme two, Challenges and enablers to care coordination, comprises four subthemes: Fragmented communication, Lack of resources, Stigma and discrimination, and Building trust and relationships.

#### 6.2.1 Subtheme 2.1. Fragmented Communication

CMHW highlighted broken communication and the need for clear communication during handover as being vital in interpersonal communication skills between ward staff and community mental health workers:

[…] I find communication can be really fragmented […] and then I'll go into ward round, and it won't reflect what has been actually handed over […] [Rose]

Another CMHW expressed her concern about the fragmented and non‐integrated care model and highlighted the need for a more cohesive approach to ensure better continuity and quality of care:

So, the fractured care model or the non‐integrated care model is where you have community consultants who do clinics all the time […]. I understand nursing staff love it because they have someone sat on the ward, more, there all the time, and they feel they have got the consultant there and they haven't got lots of consultant ward runs. [Jessica]

Many CMHWs felt that communication was a critical facilitator in the care‐coordination process, enabling efficient patient discharge. A consultant psychiatrist, Susan, emphasised the importance of effective communication with care coordinators:

So, I think the role of the care coordinator is really important. So, if you have got a care coordinator who the communication process is good with […] You can get the patient out of the hospital a lot quicker, and things work much more efficiently. [Susan]

Furthermore, a majority of CMHWs highlighted a significant barrier arising from communication issues with the ward staff:

A big barrier again is communication from the ward but again they're busy. We often ring up and get told the answer of, “We don't know.” Like if you ask about a patient's pain relief or “How were they on the ward?” the answer often is, “I don't know I've not been on shift.” So lack of communication, but I think it's more the staff are just so ridiculously busy that I can't see asking them to be more organised would change anything. I think what they just need is somebody to look at the workload on the ward or maybe more staff […]. [Hannah]

Similarly, one CMHW shared the difficulty she encountered with communication in the context of patient discharge and medication, expressing:

So that communication element I think is a challenge. And I just think, as I say, because it's almost like the last part in a patient's journey, that discharge and medication element. I think it tends to be a bit of a rush to process if I'm honest. [Rudo]

In contrast, other participants seemed to have a more positive outlook on his experiences, suggesting that he finds his role manageable without significant obstacles or difficulties:

I don't think there are too many challenges around. [Naresh].

However, the majority of CMHWs faced other challenges in coordinating patient hospital discharge, particularly concerning the scarcity of available resources in the community.

#### 6.2.2 Subtheme 2.2. Lack of Resources

CMHWs highlighted concerns about the limited availability and accessibility of resources in the community, which presented challenges in delivering adequate support and services to individuals in need:

Probably it's obviously funding issues because if I am Minister of Health, I would say give the mental health teams more funding. [Bongani]

In addition, another CMHW reported her personal and professional experiences, such as loss of client resources, such as services, benefits, and inadequate social support for patients, saying:

Right, one of the biggest challenges is over the past 13 years, no 14 years I have been working in this job, is the loss of services. Housing Benefits, everything you used to be able to get and now you can't [ …] [Hannah]

Another CMHW shared her experiences of the changes in CMHISs over the past few years. She mentioned that services used to run more smoothly and were more focused on the needs of clients. However, due to cuts in services, many resources that were once available are no longer accessible, which has affected the quality and focus of care provided to patients:

Things ran a lot better over 10 years ago. A lot of it is cuts in services. What used to be available is not available anymore. We're finding things aren't client focused anymore. Services don't seem patient‐focused […] We'll have carers saying, “I've got half an hour and if I, [noise in background] oh, and in that half an hour if I don't do what I need to do then the person afterwards won't get their care needs met.” [Hannah]

In this theme, CMHWs shared their experiences of working within different structured and culturally influenced CMHSs with one NHS organisation. They highlighted challenges related to organisational policies, limited resources, and bureaucratic procedures, which affected their ability to efficiently provide care and coordinate patient hospital discharge. Furthermore, some CMHWs found lack of resources as barriers hindering the effectiveness of care‐coordinating patient hospital discharge:

So, first thing I will say is lack of resources […] So, lack of resources for the staff, lack of resources for the patient in the sense that there is no accessibility really for services […]. [Ola]

Other CMHWs shared similar views on improving the patient discharge pathway. One CMHW suggested that the process of identifying patient needs and finding suitable accommodation should be changed to make it more efficient, especially for patients with complex needs:

I think the two things I would say is to change the way the process for you know identifying the patient needs and finding the accommodation […]. [Susan]

CMHWs described specific experiences on challenges met, including the complexities of working with patients lacking a stable place of residence, necessitating the task of securing suitable accommodation for them and supervising their treatment:

My experiences have been things like working with patients who are of no fixed abode and then having to organise appropriate accommodation for them and when people need an increase in care on discharge […] [Kate]

The experiences of resource scarcity resonated among the majority of CMHWs, highlighting a pervasive concern within the community mental health teams.

#### 6.2.3 Subtheme 2.3. Stigma and Discrimination

This subtheme underscores the need for improved education and the reduction of societal stigma surrounding mental health conditions within the community and emphasises the importance of fostering understanding and empathy. Ola and Richard mentioned the lack of education and stigma in the community as barriers to care‐coordinating patient hospital discharge.

And I will say lack of education as well about mental health condition and stigma in the community […] [Ola]

In addition, participants highlighted that certain community service providers hesitated to extend their services to individuals diagnosed with mental illness, primarily due to the presence of societal stigmatisation:

So, in this, in stigma, I guess, well stigma in mental health as in people, you know, I guess, well it is quite a broad, what is stigma in mental health? I think this idea, this negative idea of what mental health is […]. [Richard]

#### 6.2.4 Subtheme 2.4. Building Trust and Relationship

Some CMHWs shared their experiences on the challenges in building relationships with patients during the discharge process. One CMHW mentioned that the transition from the ward to the community brings other unique challenges and finds it difficult not to compare the two settings:

Yeah, Yeah, Yeah, I think it is not easy to build a rapport when we go into their own homes we are only there for an hour and to build that trust and rapport it would take so many visits […]. [Bongani]

The quotation underscores the experiences and beliefs of CMHWs, emphasising that patients' experiences of stigma can contribute to their feelings of being misunderstood.

### 6.3 Theme 3. Experiences of Organisational System and Culture

This theme explores the experiences of CMHWs on the effectiveness of their respective organisations in supporting care coordination during patient hospital discharge.

#### 6.3.1 Subtheme 3.1. Policies and Procedures

The subtheme policies and procedures focus on how participants' work is influenced by strict organisational policies and procedures that impact referrals, limited resources, and bureaucratic procedures within the broader ecological system. Participants stressed the need for policies and procedures that promote patient‐centred care, provide adequate resources, and prioritise medication safety, for example:

But with regards to bureaucracy and social, yes I think, you know, if a service is stretched, if it is understaffed for example and it's got referrals coming in left, right and centre […] they would want to be kind of […], very, very strict with the criteria of what comes into their service. [Richard]

For some CMHWs, organisational policies have had impact on CMHISs, such as issues with vacancies and staff shortages leading to increased workload for the community staff. In addition, the lack of access to other support services, such as Social Services and support groups, puts a higher demand on CMHWs to provide care and support to patients. This was illustrated by one of the CMHWs, who highlighted a combination of the above‐mentioned issues around limited resources perceived to be adding further delays in care‐coordinating hospital discharge:

Yes. I guess alongside vacancy and recruitment problems it culminates in additional workload for community staff, and you know a lot of our work is obviously around preventing admission but there are patients […]. You know Social Services lots of things closed you know access to lots of support groups […]. I think you know the demand on us was higher because other services weren't available. [Ruth]

Furthermore, participants emphasised policy and procedures stressing that discharged patients should be visited at home within 72 h:

Well in our work we have the seventy‐two‐hour follow‐ups and then if we don't see them within the seventy‐two hours, keep trying before a seven‐day breach. […] [Richard]

Several CMHWs shared their individual experiences of having difficulties with organisational procedures, especially concerning documentation and incomplete information from the ward during the coordination of patient discharge:

I think it links in a little bit with number two when I was saying from like a bureaucracy side of you know the paperwork that you would use the information that you receive from the ward may not be as detailed as you'd like it to be sometimes, and I know that's due to pressures that they face and staffing. [Mary]

#### 6.3.2 Subtheme 3.2. Team Dynamics and Leadership

This subtheme pertains to understanding how community health workers work together as a team and the role of leadership when they are helping to coordinate the discharge of patients from the hospital after receiving care. One CMHW outlined the duties assigned to them based on team dynamics and leadership:

I think my main duty and responsibilities are around safety and treatment of patients, so I think it's about working with not only the patient but with the team, with the families […]. And I think my sort of responsibilities and duties within the team are also about deciding whether somebody requires admission, what kind of support they need […] [Mary]

Whereas another consultant psychiatrist demonstrated her leadership ability by providing a detailed explanation of the functioning of the CMHISs' care model and sharing her positive experiences with its implementation, saying:

Okay. So well, where I work, here, we would use an integrated model which means that I cover a patch and my outpatients, if they need to come into hospital, I will make that decision and they are under my care in the hospital […].Occasionally, I will see people sooner if I am very worried about them. [Jessica]

### 6.4 Theme 4. CMHWs' Personal Experiences of Professional Growth

This theme highlighted the importance of personal and professional growth, focusing on the interplay between individual development, organisational support, and the broader CMHS context.

#### 6.4.1 Subtheme 4.1. Personal Wellbeing

In this study, CMHWs recognised the importance of enhancing the personal wellbeing of staff, emphasising the need for improved support and resources within the workplace for example one participant explained:

Yes, I think care for staff […] really needs improving. I think you know everybody has mental health not just our patients we all have it, […]. I think it would be beneficial for there to be more things available for staff at work […] [Mary]

Building on this, participants noted that the significant psychological drain and self‐esteem challenges experienced by CMHWs, as illustrated in the quote below:

Oh yes. So psychologically it is really, really like draining. It's draining, you lack self‐esteem as well because there's no way because you doubt yourself you find that you cannot even remember how many people that is on your caseload […]. [Ola]

#### 6.4.2 Subtheme 4.2. Continuous Professional Development and Recommendations

This subtheme focuses on how CMHWs can keep learning and growing in their field and suggestions they provided. This is important because it helps these CMHWs' narratives improve their abilities, leading to better care coordination for patient hospital discharge, and this is highlighted by the fact that the lack of continuous professional development in specialised areas is a drawback, as illustrated:

So, the other challenges include, I suppose, not having enough training in specialist areas, so because I am not nurse, I'd often have to go back to my work colleagues and ask about medication to clarify. [Julie]

Furthermore, another CMHW emphasised the significance of educating colleagues, particularly those in the wards and ward managers, about making prompt referrals. This highlights the importance of continuous learning and promoting professional growth among healthcare workers.

I've tried to educate the wards, the ward managers and a lot of the staff who work there about them making these referrals in a timely manner. [John]

Conversely, other participants expressed concerns about the lack of specialised training and support for staff wellbeing. one participant, Julie, expresses her concern about the lack of specialised training in specific areas related to her role as a community mental health worker. Julie mentions that because she is not a nurse, she sometimes feels the need to approach her colleagues to seek clarification about medication‐related matters, highlighting the importance of having more training opportunities to enhance her knowledge and skills in these specialised areas:

But certainly, the medication side. I've not got the specialist knowledge […] so that can be a barrier. [Julie]

Based on their individual experiences, the majority of the CMHWs highlighted the importance of effective communication among team members and early discharge planning to ensure a smooth and coordinated process for patient discharge. For instance:

My recommendations, I would say the key word that I can give is communication. So, communication of everybody, well to everybody who needs to know the information relating to the patient being discharged. [Rudo]

Participants recommendations emphasise that clear communication ensures all relevant parties are informed and ready, enhancing the overall discharge experience.

#### 6.4.3 Subtheme 4.3. COVID‐19 Pandemic

The COVID‐19 pandemic has had a profound impact on personal mental health, overwhelming healthcare systems, leading to significant challenges in patient care, and causing global disruptions that extended across various sectors and countries. Several participants illuminated the impact of COVID‐19. A majority of CMHWs reflected on the emotional challenges faced during the pandemic, where concerns about personal wellbeing, as well as the impact of COVID‐19, have added to the complexities of care‐coordination. One CMHW shared her experiences:

I think the pandemic has been challenging emotionally you know with care‐coordinating because we've had our own worries about that and you know our own worries about Covid […]. [Mary]

Other CMHWs shared similar experiences on the negative impact of COVID‐19. One CMHW commented:

And I think as well with the COVID‐19 been going on, relatives and carers have been unable to attend ward rounds and have their views heard. [John]

In addition, some CMHWs shared similar experiences and believed that the disruptive impact of service closures during the COVID‐19 pandemic had impacted care delivery:

Most of the services has been shut down and government are now looking into reopening it […] [Ola]

The COVID‐19 lockdown policies and regulations resulted in widespread service closures, and the government is now focused on reopening them, but limited resources hinder their efforts, making it challenging to provide necessary support and referrals for patients with a diagnosis of mental health condition in need of recovery help.

#### 6.4.4 Subtheme 4.4. Positive and Negative Personal Experiences

This subtheme highlighted both the positive and negative aspects of participants' experiences in coordinating the discharge process and supporting patients during their transition into the community:

No, it does not affect me emotionally. No, it is in terms like I say you go to work and help someone, and they are happy you feel good about yourself. [Bongani]

Some CMHWs emphasised the value of their manager's open‐door policy, characterising it as a supportive environment in which managers consistently made themselves accessible to provide help and guidance to their colleagues. One CMHW shared her remarkable experience:

Well, supervision, absolutely supervision. We've got a new Manager at our team now and she's really good and she's very keen on supervision. Her door is always open […] [Rose]

In summary, Theme 4 centred on various aspects, including personal wellbeing, continuous professional development, recommendations provided by CMHWs, experiences during the COVID‐19 pandemic, and a spectrum of positive and negative personal encounters. This theme covered ongoing efforts for professional development, including suggestions for enhancement, while also addressing the diverse personal experiences amid the challenges posed by the COVID‐19 pandemic.

## 7 Discussion

These empirical findings revealed four themes and subthemes: *Experiences of collaborative working; Challenges and enablers to care coordination; Experiences of organisational system and culture; and Community mental health workers' personal experiences of professional growth*. As illustrated in Table 2, using the Ecological System Theory (EST) and Normalisation Process Theory (NPT) lenses helps to better understand how CMHWs collaborate, overcome organisational obstacles, and care‐ coordinating discharge of patients diagnosed with mental conditions into community mental health integrated services, focusing on four key themes embedding EST with NPT.

**TABLE 2:** An application of ecological system theory embedded with normalisation process theory.

Ecological system theory levels	Key themes	Normalisation process theory constructs	Description in this study context
Macrosystem	CMHWs' experience of challenges and enablers	Cognitive Participation	CMHWs cognitively navigate, participate, and normalise challenges while working with CMHTs across all system levels.
Exosystem	Experiences of organisational system and culture	Collective Action	CMHWs actively, collectively act to sustain new working practices in organisational and cultural contexts.
Mesosystem	Experiences of collaborative working	Coherence	CMHWs make sense of their work and experiences in joint efforts across CMHTs and CMHSs.
Microsystem	CMHWs' personal experiences of professional growth	Reflective Monitoring	CMHWs and researcher engage in self‐evaluation and reflection on work practice and personal development.

*Note:* Adapted and modified from (Bronfenbrenner 1979; May et al. 2018).

**Fluid interaction:** The Ecological System Theory is not hierarchical or fixed, CMHWs may move between levels, influence across them, and experience overlapping dynamics.
**Constructs are interrelated:** Normalisation Process Theory elements like coherence or collective action do not belong to just one level; they interact fluidly with the realities of CMHWs working practice.
**Reflection and collaborative working:** Occur simultaneously across ecological system levels: CMHWs and researcher's experiences and reflections on personal and professional growth through interaction during data collection.

In the subsequent sections, underpinned by (Bronfenbrenner 1979) EST and (May et al. 2018) NPT, this discussion enables a comprehensive exploration of CMHWs' experiences of care‐coordination as illustrated in Table 2.

### 7.1 CMHWs’ Experiences of Collaborative Working

This study highlights the centrality of collaborative working in coordinating hospital discharge for individuals with mental health diagnoses. Interpreted through *Ecological Systems Theory (EST)*, collaborative working reflects processes occurring across the *mesosystem*, where interactions between community mental health teams (CMHTs), psychiatrists, social workers, general practitioners (GPs), and voluntary sector services shape the quality and continuity of care. *Normalisation Process Theory (NPT)* further illuminates these findings through the construct of *coherence*, which concerns how staff make sense of their roles and shared responsibilities within collaborative practices (May and Finch 2009).

Participants described limited opportunities for joint working, reduced communication, and fragmented relationships between CMHTs and hospital‐based teams. These experiences suggest that coherence around discharge coordination is weak, with staff struggling to develop a shared understanding of processes and expectations. This finding aligns with Forchuk et al. (2020) and Li et al. (2022), who emphasise that effective collaboration is essential for safe transitions into community settings. Similarly, Goh et al. (2022) and Petrie et al. (2021) identified persistent gaps in communication between hospital and community services, reinforcing the challenges reported by CMHWs in this study.

Barriers to collaboration were often attributed to workforce shortages, limited community resources, and inconsistent information sharing factors situated within the *exosystem* of EST, where organisational structures and resource constraints indirectly shape frontline practice. These findings echo Caplan et al. (2018) and Russell et al. (2021), who reported that insufficient service provision and poor information flow hindered multidisciplinary coordination. By integrating EST and NPT, this study demonstrates how structural pressures (exosystem) and sense‐making processes (coherence) interact to influence CMHWs' experiences of collaborative working.

### 7.2 CMHWs’ Experiences of Challenges and Enablers to Care Coordination

The experiences of CMHWs in coordinating patient discharge involve navigating multiple barriers and facilitators, reflecting the cognitive participation aspect of NPT within EST. Cognitive participation, defined by May et al. (2018) as the efforts individuals make to engage with new practices and understand work challenges, underscores the need to address systemic issues. Within the *EST framework*, these challenges span multiple ecological levels, including organisational policies (exosystem), community resources (mesosystem), and broader socio‐economic conditions (macrosystem).

Participants described significant obstacles, including staff shortages, limited community mental health services, complex patient needs, and restrictive organisational policies. These findings are consistent with Chen et al. (2021) and Russell et al. (2021), who identified similar barriers among liaison psychiatrists, nurses, and physicians. The emotional and cognitive labour required to navigate these constraints underscores the importance of cognitive participation: CMHWs must continually invest effort to maintain engagement with discharge processes despite systemic limitations.

The shift from institutional to community‐based care (Ikkos and Bouras 2021) further contextualises these challenges. As Li et al. (2022) and Nyassi et al. (2023) argue, multidisciplinary collaboration is essential for effective care coordination, yet CMHWs in this study reported frustration arising from inadequate resources and delayed referrals. These findings mirror international evidence from Ghana (Agyapong et al. 2016), the Netherlands (Beckers et al. 2019), Nepal (Brenman et al. 2014), Canada (Forchuk et al. 2020), Singapore (Goh et al. 2022), Australia (Petrie et al. 2021), the UK (Priebe et al. 2012), Malaysia (Russell et al. 2021), and Mexico (Martinez et al. 2017).

By situating these experiences within EST, the study demonstrates how ecological systems, ranging from organisational structures to national policy contexts, shape CMHWs' capacity to coordinate care. NPT complements this by explaining how staff attempt to sustain engagement with discharge processes despite these pressures. Together, these frameworks provide a coherent explanation for the complex interplay of barriers and enablers identified in the data.

### 7.3 Experiences of Organisational Systems and Culture

CMHWs' experiences of organisational systems and culture reflect processes within the *exosystem* of EST, where institutional structures, resource allocation, and organisational norms influence frontline practice. Participants described how inadequate communication pathways, limited multidisciplinary team (MDT) collaboration, insufficient community referral options, and poor accommodation availability hindered effective discharge coordination. These findings align with McEvoy et al. (2019), Murphy et al. (2018), and Russell et al. (2021), who similarly identified organisational barriers that impede coordinated care.

NPT's construct of *collective action* is particularly relevant here. Collective action refers to the operational work required to enact new practices, including the distribution of tasks, availability of resources, and organisational support (May et al. 2009). CMHWs' accounts suggest that collective action is constrained by systemic pressures such as workforce shortages, high caseloads, and limited community infrastructure. National challenges including funding constraints, recruitment difficulties, and rising demand for mental health services further exacerbate these issues (British Medical Association 2024; Hodge and Raymond 2023; Oliver 2022).

Despite these barriers, some participants reported positive experiences of collaborative working within CMHTs, indicating that supportive team cultures can facilitate collective action even in resource‐limited environments. However, persistent gaps between national policy aspirations and local service realities remain evident. Although national guidance emphasises timely discharge planning and integrated care pathways (NHS England 2019; NICE 2016; Parliamentary and Health Service Ombudsman 2024), CMHWs reported that these standards are difficult to achieve in practice.

These findings reinforce the need for organisational transformation to strengthen the *exosystem* and enable effective collective action. EST and NPT together highlight how structural constraints and operational work interact to shape CMHWs' experiences of discharge coordination.

### 7.4 CMHWs’ Personal Experiences and Professional Growth

CMHWs' reflections on their personal experiences and professional development illustrate processes occurring within the *microsystem* of EST, where individual beliefs, skills, and interpersonal relationships shape practice. Through the lens of NPT, these experiences relate to *reflexive monitoring*, the appraisal work individuals undertake to evaluate and adapt their practices (May and Finch 2009).

Participants described how coordinating complex discharges fostered professional growth, enhanced confidence, and deepened their understanding of their roles within community mental health services. These findings contribute to a limited body of qualitative research exploring CMHWs' perspectives, particularly during and after the COVID‐19 pandemic (Liberati et al. 2021). While much existing literature focuses on patient experiences especially barriers to access and stigma (Caplan et al. 2018) this study highlights the importance of understanding CMHWs' experiences to inform community mental health service improvement.

Organisational improvements are essential to strengthen CMHWs' personal and professional growth to improve care coordination within community mental health services. These recommendations align with WHO guidance and the NHS Long Term Plan, which emphasise strengthening community mental health provision and improving continuity of care (NHS 2019a, 2019b; NHS England 2019, 2023; WHO 2018). However, participants reported that discharge coordination in the UK remains inconsistent and often insufficient compared with international models of integrated, neighbourhood‐based care (Castillo et al. 2019; Ejegi‐Memeh et al. 2025; Obegu et al. 2025).

By integrating EST and NPT, this study provides a conceptual framework that explains how individual, organisational, and systemic factors shape CMHWs' experiences of discharge coordination. These findings underscore the need for systemic change to support staff, enhance collaborative working, and improve patient outcomes.

## 8 Trustworthiness

Trustworthiness is a cornerstone of qualitative research and is essential for ensuring credibility, transferability, dependability, and confirmability of findings (Adler 2022; Squires and Dorsen 2018). This was maintained through researcher reflexivity and the use of an audit trail, supporting auditability and replicability by documenting the research process for future use (Yadav 2021). An insider perspective further enhanced trustworthiness and rigour by providing deep contextual understanding and establishing rapport with participants, facilitating rich and meaningful data generation.

## 9 Strengths and Limitations of the Current Study

A key strength of this study is the application of EST and NPT, which provides theoretical insight into the ecological factors influencing CMHWs' effectiveness in hospital discharge processes. Limitations include the single NHS Trust setting and a small sample size, which may restrict transferability (Vasileiou et al. 2018). Additionally, in relationship to the study findings, the overrepresentation of female and White British participants may introduce bias (see Table 1). The shift to online interviews due to COVID‐19 may also have affected data quality by limiting the observation of nonverbal cues (Carter et al. 2021; de Villiers et al. 2021).

## 10 Conclusions

CMHWs across the Trust highlighted similar challenges from their perspectives. Interestingly, CMHWs' experiences were across all CMHTs. This study's findings call for a change in working practices, signal a pivotal moment in my journey towards improving the professional working environment for CMHWs. Based on the findings, the study contributes to our understanding of varied experiences of how CMHWs navigate these intricate environments. In addition, this study has revealed that CMHWs experience tensions and challenges, yet also opportunities for growth and resilience.

## 11 Relevance for Implication to Clinical Practice

Based on the findings of this study, several practice, policy, and research recommendations are proposed. Community mental health workers should be more formally integrated into hospital discharge pathways through standard care protocols that promote cohesive, patient‐centred coordination between inpatient and community mental health services. To address fragmented communication and inconsistent handover practices, standardised digital hospital discharge summaries shared across inpatient and community mental health teams should be implemented, complemented by brief daily cross‐team huddles during high‐risk discharge periods to enable real‐time information exchange. In response to workforce pressures, community mental health teams should introduce protected time for caseload review and establish minimum staffing thresholds during peak discharge activity, supported by workforce‐modelling tools that account for clinical complexity rather than headcount alone. To address identified training gaps, particularly in relational risk assessment and interagency negotiation, mandatory joint training for inpatient and community mental health workers is recommended, incorporating simulation‐based learning and structured reflective practice. Further research is needed to evaluate the effectiveness of these interventions and to strengthen the evidence base informing community mental health practice and policy related to care coordination and discharge planning.

## Funding

The authors have nothing to report.

## Conflicts of Interest

The authors declare no conflicts of interest.

## Data Availability

The data that support the findings of this study are available on request from the corresponding author. The data are not publicly available due to privacy or ethical restrictions.
